# The potential role of lactulose pharmacotherapy in the treatment and prevention of diabetes

**DOI:** 10.3389/fendo.2022.956203

**Published:** 2022-09-15

**Authors:** Natural Chu, James Ling, He Jie, Kathy Leung, Emily Poon

**Affiliations:** Department of Medicine and Therapeutics, Chinese University of Hong Kong, Prince of Wales Hospital, Hong Kong, Hong Kong SAR, China

**Keywords:** medication, short-chain fatty acids, diabetes, microbiota, hormones

## Abstract

The non-absorbable disaccharide lactulose is mostly used in the treatment of various gastrointestinal disorders such as chronic constipation and hepatic encephalopathy. The mechanism of action of lactulose remains unclear, but it elicits more than osmotic laxative effects. As a prebiotic, lactulose may act as a bifidogenic factor with positive effects in preventing and controlling diabetes. In this review, we summarized the current evidence for the effect of lactulose on gut metabolism and type 2 diabetes (T2D) prevention. Similar to acarbose, lactulose can also increase the abundance of the short-chain fatty acid (SCFA)-producing bacteria *Lactobacillus* and *Bifidobacterium* as well as suppress the potentially pathogenic bacteria *Escherichia coli*. These bacterial activities have anti-inflammatory effects, nourishing the gut epithelial cells and providing a protective barrier from microorganism infection. Activation of peptide tyrosine tyrosine (PYY) and glucagon-like peptide 1 (GLP1) can influence secondary bile acids and reduce lipopolysaccharide (LPS) endotoxins. A low dose of lactulose with food delayed gastric emptying and increased the whole gut transit times, attenuating the hyperglycemic response without adverse gastrointestinal events. These findings suggest that lactulose may have a role as a pharmacotherapeutic agent in the management and prevention of type 2 diabetes *via* actions on the gut microbiota.

## Introduction

### Applications of lactulose

Lactulose is a synthetic disaccharide (C_12_H_22_O_11_) derived from lactose using β-galactosidase or epimerase that is most commonly used as a laxative agent. It consists of fructose and galactose. Lactulose is poorly absorbed from the gastrointestinal tract, and there is no digestive enzyme capable of hydrolysis in cells lining the intestinal tract. Therefore, lactulose entering the large intestine remains virtually unchanged and is then fermented by gut microbiota that increase osmotic effects and intraluminal gas formation. It increases stool amount and softens stool, eliciting its laxative effect to treat subjects with constipation. However, such biochemical and physiologic activities can cause borborygmi, bloating, belching, flatulence, and diarrhea. Nevertheless, its laxative effect in the management of chronic and functional constipation is less efficacious than the use of polyethylene glycol. Apart from being used as a stool softener, lactulose is also used to detect small intestinal bacterial overgrowth during a hydrogen breath test.

The use of lactulose may not be limited to a laxative agent. Lactulose is routinely used in the management of hepatic encephalopathy (HE), which involves the accumulation of nitrogenous waste products in the systemic circulation (1). The formation of lactic acid and small amounts of acetic and formic acids results in an acidic environment in the gut, with resultant volatile fatty acid metabolites leading to a decrease in the permeability of the gut membrane (2). Under these situations, ammonia diffuses into the acidic colon and is ionized into ammonium ions, facilitating the excretion of excess ammonia. Furthermore, the gut microbiota plays an essential role in the pathogenesis of HE, and the routine treatment strategy is directed to modulate intestinal microbiota profiles, short-chain fatty acids (SCFAs), and their function by the administration of the non-absorbable disaccharide lactulose. In HE, lactulose serves as a prebiotic rather than a laxative. In a randomized controlled multicenter trial involving 98 cirrhotic patients, there were significant differences between the lactulose and control groups in the abundance of *Actinobacteria*, *Bacteroidetes*, *Firmicutes*, and *Proteobacteria* (3).

In addition, lactulose may be useful in the management of chronic kidney disease (CKD). In a prospective interventional trial involving 40 patients with stage 3 and 4 CKD (4), lactulose reduced the nitrogen products and urea, creatinine, uric acid, and β2-microglobulin levels. Lactulose also suppressed tubulointerstitial fibrosis in a rat model of a kidney disease study (5). Lactulose modifies the gut microbiota, increasing the abundance of *Bifidobacteria* and *Lactobacilli* after 8 weeks of 30 ml lactulose syrup thrice daily, and ameliorates CKD progression by suppressing uremic toxin production (5, 6). Moreover, lactulose can minimize the formation of gallstones by increasing the intestinal transit time, which influences the anaerobic bacterial enzymatic biotransformation of conjugated cholate secreted by the liver to more hydrophobic deoxycholate (7). This process favors a subsequent reduction in secondary bile acid formation (deoxycholic acid), which is a metabolic by-product of intestinal bacteria, and cholesterol saturation of the bile (8). Furthermore, Lactulose has prebiotic effects that have been proven to reduce fasting and postprandial glucose and inflammation markers and improve insulin sensitivity and lipid profile in subjects with prediabetes (9).

Those underlying mechanisms are helping us to recognize the background information of lactulose pharmacotherapy and how these actions could be beneficial in controlling and preventing the development of type 2 diabetes (T2D). In this article, we listed the effect of lactulose on glucose response, the similarity of lactulose and acarbose (common glucose-lowering drug), the orchestration of health-promoting microbiota highlighted in diabetes, and the possible mechanisms involved in the treatment and pathogenesis of diabetes.

Based on published studies, lactulose may act as a prebiotic, which has positive effects in preventing and controlling diabetes (10). Prebiotics can influence gut–systemic metabolism interactions involved in the pathogenesis of diabetes. As a prebiotic, lactulose may act as a bifidogenic factor with positive effects in preventing and controlling diabetes. In this review, we summarized the current evidence for the effect of lactulose on gut metabolism and type 2 diabetes prevention.

### Search strategy

For the PubMed library, combinations of controlled terms (MeSH and Emtree) and keywords were used whenever possible, and other terms not indexed as MeSH and filters were also applied. The key terms used were as follows: (“lactulose” [MeSH Terms]) OR “prebiotics”, [MeSH Terms]) AND “prediabetes” [MeSH Terms]) OR “IGT” [MeSH Terms]) OR “diabetes” [MeSH Terms]) OR “diabetes complications” [MeSH Terms]) OR “diabetes mellitus, type 2” [MeSH Terms]) OR diabetes [Title/Abstract]) OR diabetic [Title/Abstract]). The retrieval methods for the other databases were adjusted appropriately based on the above terms. Studies were included if they were randomized controlled trials (RCT) conducted in humans and animals that were related to prediabetes and diabetes or glucose and insulin. Studies were excluded if they used lactulose for motility test or hydrogen breath diagnosis test.

### Effect of lactulose on glucose response

The effect of lactulose on metabolism and the possibility of using it to reduce blood glucose level have been examined in patients with type 2 diabetes. In 10 obese patients studied on two consecutive days controlled for an equicaloric part of diets, 1 day was randomized to 8.2 g of lactulose (equivalent to 12.2 ml of lactulose). The average glucose level of supplements with lactulose decreased by 0.53 ± 0.28 mmol/L, and insulin decreased by 74.6 ± 45.2 pmol/L (11). A similar short-term effect was shown in another crossover study involving 10 obese patients who response to 8.25g of lactulose, plasma glucose and insulin levels were reduced when compared to the placebo diet (12). Lactulose was found to reduce plasma lipopolysaccharide (LPS) endotoxin *via* changing the gut diversity as shown in an animal study (13) and increase insulin sensitivity (14). In another feeding study on 10 healthy volunteers who were given high/low glycemic index meals with or without lactulose, respectively, in a random sequence, the undigested carbohydrate lactulose, independent of its effect on a food’s glycemic index, had reduced postprandial responses and delayed gastric emptying (15). In order to investigate the short-term effect of lactulose prescription, a randomized, crossover, controlled study was conducted on 10 patients with insulin resistance and coronary heart disease given lactulose treatment for 6 days, and it was found that there were no significant differences in habitual diet with lactulose on insulin, glucose, free fatty acids, and glucagon-like peptide 1 (GLP1) response when compared to the habitual diet alone (16). The authors indicated that there was a possibility that the oral glucose tolerance test (OGTT) was not sensitive to capture the short-term effect of postprandial glucose and insulin change, and an improvement in the recent technology of continuous glucose monitoring and glucose clamping can be useful for capturing the change of incremental glucose area-under-the-curve. However, there is no long-term effect on lactulose feeding study in subjects with prediabetes or type 2 diabetes. In a recent randomized, crossover, controlled study on 24 healthy volunteers, they were randomized to receive 10 or 20 g of lactulose, 20 g of glucose, and 250 ml of water (as a negative control) separated by a 7-day washout period. There was no difference in the blood glucose profiles and calculated pharmacokinetic parameters when lactulose was given in different dosages (17). In another similar study design, 24 patients with type 2 diabetes were randomized to receive a single dose of 20 or 30 g of lactulose, 250 ml of water, and 30 g of glucose (as a positive control). The intake of both 20 and 30 g of lactulose doses resulted in a slight net decrease in blood glucose concentrations of approx. −0.3 mmol/L from baseline as assessed by an overall negative area under the curve (AUC) of postprandial glucose response (18). Both studies showed that the oral intake of a single dose of lactulose remained largely stable in the blood glucose concentrations despite a continuous fasting period of 3 h either in healthy subjects or patients with T2D. These human studies are summarized in **Table 1**. In an animal study, the administration of a single-dose lactulose was observed to have a 40% reduction in glucose absorption in the jejunal loop when compared to saline (19). Nonetheless, no study has investigated the frequency of dosage effect of lactulose on subjects with prediabetes or diabetes. For example, in the treatment of HE, lactulose is typically administered orally at a dose of 15 to 30 ml two to four times a day (20).

**Table 1:** The summary of lactulose on the effect of glucose response in human studies.

Drugs	Author (year)	Subjects	Periods	Study design	Outcomes	Additional remarks
Lactulose 20 and 30 g (equivalent to 30 and 45 ml)	Pieber et al., 2021 (18)	24 type 2 diabetes	Single dose	Randomized, controlled, crossover study	No significant differences in glucose iAUC between lactulose and water in glucose were observed, but there was a slight decrease in net glucose iAUC either in 20 and 30 g of lactulose from baseline.	No supplement with foods
Lactulose 10 and 20 g (equivalent to 15 and 30 ml)	Steudle et al., 2018 (17)	24 healthy volunteer	Single dose	Randomized, controlled, crossover study	No significant differences in glucose iAUC between lactulose and water in glucose were observed.	No supplement with foods
Lactulose 5 g (equivalent to 7.5 ml)	Brighenti et al., 2006 (15)	10 healthy volunteer	3 test meals (high GI, high GI with lactulose, and low GI)	Randomized controlled feeding study	Fermentable dietary fiber in low GI meals and high GI meals with lactulose improved glucose tolerance in the next meal and lowered the non-esterified fatty acids and delayed gastric emptying.	Supplements with foods
Lactulose 15 and 30 g (equivalent to 22.5 and 45 ml)	Frost et al., 1999 (16)	10 insulin-resistant patients with coronary heart disease	6 days of lactulose loading (15 g/day for 2 days and 30 g/day for 4 days).	Randomized, controlled, crossover study	No significant effect on insulin, glucose, free fatty acids, and glucagon-like peptide 1 (7–36) response in patients with coronary heart disease.	Supplement with habitual diet
Lactulose 8.2 g (equivalent to 12.2 ml)	Bianchi et al., 1997 (11)	10 obese patients	1-day diet with lactulose vs. 1-day placebo diet	Randomized controlled feeding study	Lactulose decreased the average glucose level by 0.53 ± 0.28 mmol/L and insulin by 74.6 ± 45.2 pmol/L.	Supplements with foods
Lactulose 8.25 g (equivalent to 12.4 ml)	Bianchi et al., 1994 (12)	10 obese patients	1-day diet with lactulose vs. 1-day placebo diet	Randomized controlled feeding study	Glucose level was reduced and insulin response was halved when compared to the placebo diet, and the insulin response to meals was blunted by 100–250 pmol/L.	Supplements with foods

iAUC, incremental area under the curve; GI, glycemic index.

### The possible pharmacological mechanism of lactulose on diabetes

**Figure 1:**
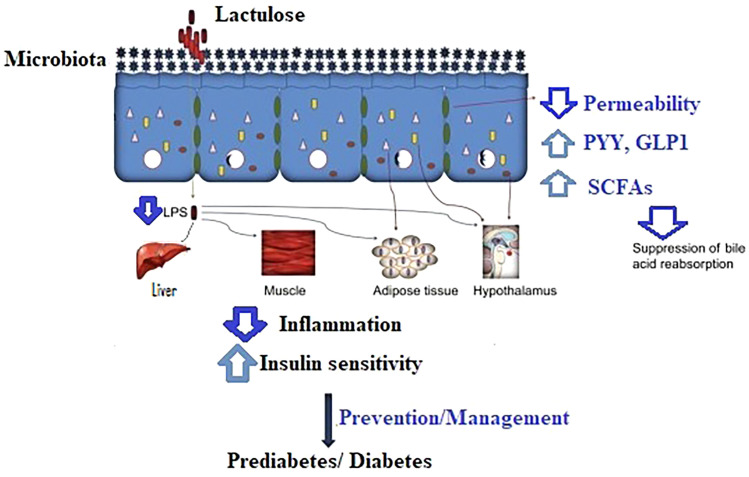
The proposed mechanism of lactulose in the prevention/management of prediabetes and diabetes. LPS, lipopolysaccharides; PYY, peptide tyrosine tyrosine; GLP1, glucagon-like peptide 1; SCFAs, short-chain fatty acids.

Lactulose acts as non-digestible carbohydrates similar to other dietary fermentable carbohydrates, such as resistant starch and oligosaccharides. They interact with the gut microbiota and promote the production of SCFAs (21), which affect local and remote motility of the gastrointestinal tract as a consequence of stimulating gastric emptying and hormone release [ghrelin (22), peptide tyrosine tyrosine (PYY) (23), and GLP1 (24)], which needs to be taken into account in using pharmacological stimulants for the prevention of diabetes in controlling glucose homeostasis. **Figure 1** depicts the interactions of lactulose and metabolism in the body.

#### The similarity of glucose-lowering drug acarbose and lactulose

The liver is the major organ for the distribution, storing, and detoxification of nutrients absorbed from the gastrointestinal tract, and abnormal liver function leads to adipose tissue accumulation, dysregulation of waste products, and excessive storage of nutrients. Consequently, it develops into liver diseases, such as non-alcohol fatty liver disease (NAFLD), HE, and cirrhosis. Lactulose is the first-line treatment for HE as previously mentioned. The current medical management of HE involves the prescription of lactulose as well as acarbose (25). Alpha-glucosidase inhibitors represent well-known oral antidiabetic agents used for the treatment of T2D. The most widely used compound, acarbose, also inhibits salivary and pancreatic amylase, and it is believed that acarbose primarily lowers postprandial glucose excursions by delaying the rate of carbohydrate digestion and absorption, allowing an excessive amount of undigested carbohydrates to reach the colon. In general, acarbose is used to turn digestible carbohydrates into poorly absorbed and fermentable short-chain carbohydrates that reduce the absorption of excessive macronutrients, and lactulose itself is an artificial indigestible short-chain fermentable carbohydrate that exerts a similar effect to acarbose-induced products for the interaction of gut microbiota and other mechanisms including SCFA promotion, gut hormone production, and reduction of secondary bile acids. Moreover, the amount of lactulose that enters the large intestine can be fixed together with the amount of oral lactulose applied, but acarbose depends on the amount of habitual carbohydrate intake (26). The addition of the respective carbohydrates fermented by the gut microbiota induced the growth of intestinal bacteria that are beneficially related to the host’s health. A higher concentration of these beneficial SCFAs as well as a higher ratio between primary bile acids and secondary acids in the lumen was found, implying a lower level of inflammation (27, 28). Acarbose is a drug proven to be efficacious and safe in the Chinese population with prediabetes and at different stages of diabetes. Current evidence has strongly reflected that acarbose manages diabetes *via* the modification of the gut microbiota, and *Lactobacillu*s and *Bifidobacterium* are enriched in the fecal samples from T2D participants after a single use of acarbose (28). Meanwhile, lactulose orchestrates the pattern of the gut microbiome with an increase of the SCFA-producing bacteria *Bifidobacterium* and *Lactobacilli* (29). Lactulose and acarbose shared a similar mechanism in the treatment of diseases *via* the modification of the gut microbiota. Furthermore, acarbose decreased blood ammonia, postprandial blood glucose, C-peptide levels, and glycated hemoglobin values in 107 patients with HE and T2D when compared to placebo randomized each for 8 weeks (30). Therefore, lactulose may create acarbose effects and increase glucose tolerance in patients with diabetes. However, only limited studies had evaluated the use of lactulose for the treatment of T2D.

#### Effect on the gut microbiota after the administration of lactulose

In general, many studies confirm the ability of lactulose to stimulate the growth of the health-promoting bacteria *Bifidobacteria* (31) which influence the metabolism. Dysbiosis is normally a common feature of prediabetes and type 1 and type 2 diabetes (32, 33). Dysbiosis refers to an imbalance between the types of microbiota present in a host gut which may contribute to a range of conditions and illnesses. The administration of fermentable carbohydrates can decrease the progression of diabetes when an increase of SCFAs and an abundance of health-related bacteria are found. In a large-scale, fecal analysis involving 304 healthy subjects after the administration of lactulose (3–5 g/day), the microbial population of *Bifidobacteria* was significantly increased and the potentially pathogenic bacteria *Bacteroidaceae*, *Eubacteria*, and *Clostridia* were decreased (34). In another study conducting a fecal analysis of 10 healthy volunteers before and after receiving 10 g of lactulose weekly, the abundance of *Bifidobacterium*, *Lactobacillus*, and *Enterococcus* spp. was induced (35). In a longer period of receiving 3 g of lactulose for 2 weeks with habitual diets, the relative abundance of *Bifidobacterium* increased significantly, and *Clostridium perfringens* and *Bacteroidaceae* decreased slightly when compared to the baseline. Apart from these, the most important modifications in the gut microbiota were found in one of the animal studies: an increase in the obesity- and diabetes-related bacteria strain *Akkermansia* and a decrease in the hyperglycemia-related bacteria strain *Desulfovibrionaceae* (33) after the lactulose intervention (36). *Akkermansia* was found to control the essential regulatory system of glucose and energy metabolism (37, 38). Similar results were observed in animal studies: 0.1% and 0.2% of lactulose were fed, the health-promoting bacteria *Lactobacillus* was increased, and the potential pathogenic *Escherichia coli* (39) was reduced in weanling pigs after 10 days and in broilers after 28 (40) and 35 days (41). Interestingly, as mentioned, polyethylene glycol has a stronger laxative effect than lactulose, but only lactulose was found to induce the abundance of *Bifidobacteria* and another anaerobe in a single-blinded RCT when compared to polyethylene glycol. Of note, polyethylene glycol inhibited the metabolic activity of the gut microbiota with a significant reduction of SCFAs (42).

Considering the results of clinical studies published to date, the induction of SCFA-producing bacteria after the administration of lactulose was similar to the current glucose-lowering drug acarbose. In both animal and human studies, acarbose promoted the abundance of *Bifidobacterium* and inhibited the abundance of *Bacteroides* (33). On further investigation at the species level, *Bacteroides plebeius*, *Bacteroides dorei/vulgatus*, and *Clostridium bolteae* were observed after the administration of acarbose. A comparison of the microbiome by lactulose and acarbose is listed in **Table 2**.

**Table 2:** Medications (lactulose and acarbose) and effects on the microbiome at the genus and species levels in healthy subjects or diabetic patients.

Lactulose	Acarbose
At the genus level:↑ Lactobacillus and Bifidobacterium↓ Bacteroidaceae, Eubacteria, and ClostridiaAt the species level:↑ Bifidobacterium longum/breve and Akkermansia↓ Clostridium perfringens, Desulfovibrionaceae, and Bacteroidaceae	At the genus level:↑ Lactobacillus and Bifidobacterium↓ Ruminococcus, Butyricicoccus, and Phascolarctobacterium At the species level:↑ Bifidobacterium longum↓ Bacteroides plebeius, Clostridium bolteae, and Bacteroides dorei/vulgatus

↑, increase; ↓, decrease.

#### Mechanisms by which lactulose can improve glucose metabolism

##### Effect on SCFAs and secondary bile acids

SCFAs induced by the fermentation of lactulose nourish mucosal cells, enhance hepatic gluconeogenesis and lipogenesis (43), and reduce secondary bile acids, influencing the renal handling of uric acid (4). These health implications are significant in terms of modifying the risk factors for the development of diabetes, and preventing the metabolic effects of the non-absorbable carbohydrate lactulose is likely to contribute to immune regulation and systemically to metabolism (44, 45). SCFAs activate G-protein-coupled receptors on enteroendocrine L cells which strengthen the gut barrier function, in regulating gut permeability and pH, primarily *via* orchestration of the tight junction proteins that prevent the translocation of LPS endotoxins resulting in the reduction of the inflammatory response (46). Therefore, minimizing the production of endotoxins has an antidiabetic effect (13). This process is also favorable with the subsequent reduction in deoxycholic acid (secondary bile acid) formation and cholesterol saturation of bile. Bile acid profiles influence the development of T1D and T2D progression. Both types of diabetes have lower levels of the primary bile acid and higher levels of the secondary bile acid profiles (47). Modulation of secondary bile acid concentrations results in a protective effect on pancreatic β cells and reserves systemic insulin load (48, 49).

##### Effect on gut hormones and gastrointestinal motility

Several hormones are vital for the control of meal size, meal timing, and meal-related glycemia. Lactulose induces the suppression of ghrelin (22) that is responsible for hunger sensation and appetite and the production of GLP1 and PYY *via* the simulation of SCFAs. PYY is known to inhibit the motility of the upper digestive tract and restores impaired insulin and glucagon secretion in pancreatic islets through the activation of hypothalamic neuropeptide Y2 receptors (NPYR2) (50), indicating that SCFAs can affect motility at a distance from their site of production. Naive GLP1 is distributed in the small and large intestines, and the augmentation of GLP1 results in the improvement of beta-cell function in fasting and postprandial hyperglycemia (51). Recently, the analogs of human GLP1 (liraglutide) have been developed into a new class of glucose-lowering drugs called ubiquitous dipeptidyl peptidase (DPP)-4 (52). These hormones diffuse from the mucosa in the gastrointestinal tract into mesenteric capillaries, which drain into the hepatic portal vein and end in synapse-like appositions to glial cells of the enteric nervous system and other cell types. These responses activate the brain–gut axis and regulate gastrointestinal motility, and these motility responses induced by lactulose reduced gastric tone (23) and delayed gastric emptying (53).

Lactulose may also stimulate tonic and phasic pyloric pressures and reduce antral and duodenal pressure waves during intestinal peristaltic activity. In an animal study, lactulose increased the small intestinal transit time but decreased the total whole gut transit time (54). In a human study of 10 healthy volunteers who consumed standard meals with 12 g of solid or liquid lactulose or inulin with 5-day washout periods separately, liquid lactulose resulted in shorter orocecal transit time than solid lactulose, but both delayed gastric emptying and accelerated the gut transit of food in comparison to inulin (a longer chain of fermentable carbohydrates) (53). These factors affect small intestinal nutrient transport related to meal-related glycemia in control of the permissive cause of overabsorption. Changes in GI hormone secretion of lactulose pharmacotherapy provide plausible mechanisms for the therapeutic efficacy in the treatment of obesity and diabetes.

#### Hydrogen production *via* fermentation of lactulose and oxidative stress

Diabetes complications are associated with higher reactive oxygen species (ROS) production in cells, which is an important mediator for the activation of proinflammatory signaling pathways. Obesity and hyperglycemia-induced ROS production including superoxide (*O*_2_), hydrogen peroxide (H_2_O_2_), and hydroxyl radical (OH^•^) were linked to metabolism and inflammation in diabetes and macro/microvascular complications (55). Excessive ROS activates proinflammatory transcription factors such as NF-κB and AP-1 that upregulate the expression of cytokines (56, 57). In an animal study of 18 rats that were given 5.3 g/kg of lactulose, ROS and cytokines were measured, and lactulose repressed significantly the overexpression of free radicals and multiple cytokines, such as NF-κB, IL-1β, and IL-6 (58). This was through increased production of endogenous hydrogen gas (H_2_) by the fermentation of lactulose in the gut (59). Lactulose led to a higher production of the non-functional gas H_2_ than other natural short-chain fermentable carbohydrates, such as lactose and fructose (1). H_2_ is defined as a selective antioxidant that can selectively neutralize toxic free radicals in the body (59). In a human study, 102 obese and/or diabetic subjects were enrolled, and a free oxygen radical test was conducted to evaluate the ROS level (60). Unsurprisingly, the obese/diabetic subjects had higher free radical values (H_2_O_2_) than non-diabetic subjects, and these values were positively correlated to fasting glucose, total cholesterol, body mass index, and uric acid. In an animal study, cytokines (TNF-α, IL-1β) and oxidative stress (maleic dialdehyde and marrow peroxidase) in the colon were suppressed by lactulose administration and intraperitoneally injected H_2_-rich saline *via* increasing endogenous H_2_ production (61). From these observations, lactulose can be a ROS scavenger to suppress oxidative stress in diabetes complications.

#### Weight loss

Impaired intestinal permeability is a feature of obesity, diabetes, and associated metabolic disorders (62). Higher intestinal permeability assessed by the lactulose:mannitol ratio was found in obese subjects in a prospective interventional 1-year weight loss intervention. Intestinal permeability was highly correlated to the fasting blood glucose level in this study (63). In another animal study, lactulose was found to increase the body weight gain but not organ weight in broilers after 35 days of lactulose intervention (41). Moreover, this observation was also found in dextran sodium sulfate-induced colitis mice that were fed with lactulose for 7 days, and body weight loss was reduced when the administration of oral lactulose was increased (61). In a human study, 76 Japanese women were randomized to receive 4 g of lactulose with 300 mg of calcium and 150 mg of magnesium or as control over 12 months. There were no differences in anthropometric data (e.g., weight loss and waist circumference) between the two groups, but there was a trend of lower body fat percentage after the intake of lactulose (64). Nevertheless, the *in-vivo* treatment with lactulose of high fat-induced rats for 8 weeks significantly alleviated body weight gain, plasma free fatty acids, triglyceride, leptin, and insulin level when compared to the control groups, but it had no influence on liver function, such as aspartate aminotransferase (AST) and alanine aminotransferase (ALT). Furthermore, lactulose downregulated the expression levels of major adipogenic proteins (C/EBPα and PPARγ) and stimulated oxidative phosphorylation, β-oxidation, and thermogenesis, stimulating energy expenditure and lipolysis (65).

### Adverse effects of lactulose

#### Gastrointestinal side effects

Gastrointestinal side effects are common with metformin and acarbose which are often used as first-line treatment. Patients with diabetes sometimes discontinue these drugs due to intolerable gastrointestinal symptoms. These side effects are dose-dependent. Lactulose rarely causes serious adverse effects (66), and the most common gastrointestinal symptom is bloating (67). Some studies suggest that a low dose of 1–3 g of lactulose daily for 2 weeks may be tolerated by most people without significant gastrointestinal side effects (68). The studies below demonstrate that low-dose lactulose (1–10 g/day) is well-tolerated in healthy adults and those with mild constipation and mild gas-related gastrointestinal symptoms (**Table 3**). However, there was no study that investigated the gastrointestinal effects of lactulose use in prediabetes and diabetes.

**Table 3:** Studies of low-dose lactulose on gastrointestinal effects.

Drugs	Author (year)	Subjects	Durations	Study design	Outcomes
Lactulose (2 g/day)	Sakai et al., 2019 (69)	52 healthy adult volunteers	2 weeks	Randomized, double-blind, placebo-controlled, crossover study	Lactulose was well-tolerated and no serious adverse events were reported.
Lactulose (1, 2, and 3 g/day)	Sakai et al., 2019 (68)	26 healthy adult volunteers	2 weeks	Open-label, single-arm, before–after study	Lactulose was well-tolerated and no serious adverse events were reported.
Lactulose (10 g/day)	Bouhnik et al., 2004 (70)	16 healthy adult volunteers	6 weeks	Randomized, double-blind, placebo parallel controlled study	Lactulose was generally well-tolerated, and gas-related symptoms were observed but there were no significant differences between the placebo groups.
Lactulose (3–5 g/day)	Mizota et al., 2002 (34)	304 subjects (one group non-constipated; one group mildly constipated)	2 weeks	Open-label study	Lactulose was generally well-tolerated at all doses, and only gas-related symptoms (distension and flatulence) were slightly increased.
Lactulose (10 g/day)	Tuohy et al., 2002 (71)	20 healthy adult volunteers	8 days	Randomized, double-blind, placebo-controlled study	Lactulose was generally well-tolerated, but one subject reported a moderate to a severe change in flatulence, bloating, and abdominal pain.

#### Other adverse effects

Additionally, individuals who abuse laxatives may be promulgated on certain beliefs that daily bowel movement is necessary for good health and weight loss. However, there are medical problems involving the changes in electrolytes and acid/base that can involve the renal and cardiovascular systems. The causes of weight loss are mainly due to water depletion (72). There is evidence from isolated reports that Lactulose might influence the absorption of vitamin K from the gut and thus potentiate the warfarin treatment (73). However, there was no prescription detail of lactulose in the report, such as the duration and dosage. Further investigation on the interaction between warfarin and lactulose should be conducted. Hypovitaminosis is associated with impaired intestinal mucosal permeability, and fermentation of lactulose will decrease the pH of the gut and reduce the intestinal permeability (74). Therefore, a low dose of lactulose could not affect T2D patients with hypovitaminosis. This case report was rejected by the single-blind, randomized, multicenter, and parallel-group comparative study of 245 elderly with a history of constipation. There were no significant differences in nutritional parameters including albumin, ferritin, and vitamins and in the evaluation of denutrition risk using the Mini Nutritional Assessment after 6 months of lactulose (10–30 g/day) treatment when compared to the baseline (75). Disruption in the absorption of vitamins when a patient has an impaired gut would be one of the reasons for the loss of vitamins and other functional nutrition ingredients. These nutritional states are influenced by other diseases such as inflammatory bowel disease (IBD) and may also be influenced by the consumption of a habitual diet that contains natural prebiotics and other fermentable carbohydrates in foods (76). Furthermore, a low dose of lactulose can increase the absorption of minerals. In a double-blind, randomized, crossover study involving 24 healthy subjects, 2 or 4 g of lactulose, 300 mg of calcium, and 150 mg of magnesium were labeled using stable isotopes. A higher dose of lactulose increased the urinary stable isotope ratios of calcium and magnesium (77, 78). A significant difference in calcium absorption between 10 g of lactulose and placebo was shown which exerts a prebiotic effect in 12 postmenopausal women for a 9-day consumption (79). A similar result was also shown in an animal study, where Ca absorption was stimulated when lactulose, as well as other poorly digestible carbohydrates, such as raffinose and sorbitol, was administrated (80, 81). Hypernatremia may occur when very high doses of lactulose are given (>100 ml/day) (82). A low dose (1–10 g/day) of lactulose is well-tolerated in a study population (**Table 3**) without any severe adverse events even in the elderly group (83).

Therefore, a low dose of lactulose in the short term may not affect hyperuricemia, hyperaldosteronism, and other electrolyte and mineral changes.

## Conclusion

Here, we summarized the data confirming the possibility of using non-absorbable disaccharides such as lactulose as a pharmacotherapeutic agent for diabetes, through an increase of gut permeability due to the acidic gut environment, SCFA formation, mineral absorption, secondary bile acids, ROS reduction, and orchestration of the gut microbiota. A low dose of lactulose is safe and can be used in combination with probiotics or other prebiotics for the treatment of prediabetes and the prevention of diabetes (9, 84), which may reduce the inflammatory response and oxidative stress in diabetes (85, 86). Beneficial probiotic strains such as *Lactobacilli* and *Bifidobacteria* can produce bioactive isomers of linoleic acid and reduce the proinflammatory gene, having antidiabetic properties (87, 88). However, lactulose may have potential adverse effects including interruption of vitamin and mineral absorption in the long term. Lactulose shows benefits to the gut microbiota and host immunity, but the long-term applications of lactulose are still pharmacologically evaluated. The potential of lactulose as a pharmacotherapy for prediabetes and diabetes needs to be formally evaluated in larger randomized controlled studies across populations.

## Author contributions

NC designed the study and wrote the manuscript with support from KL and HJ. JL and EP worked on the manuscript and proof of outline. All authors contributed to the article and approved the submitted version.

## Conflict of Interest

The authors declare that the research was conducted in the absence of any commercial or financial relationships that could be construed as a potential conflict of interest.

## Publisher’s note

All claims expressed in this article are solely those of the authors and do not necessarily represent those of their affiliated organizations, or those of the publisher, the editors and the reviewers. Any product that may be evaluated in this article, or claim that may be made by its manufacturer, is not guaranteed or endorsed by the publisher.
